# Study of the sustained speed of kill of the combination of fipronil/amitraz/(S)-methoprene and the combination of imidacloprid/permethrin against *Dermacentor reticulatus*, the European dog tick

**DOI:** 10.1051/parasite/2011184319

**Published:** 2011-11-15

**Authors:** J.J. Fourie, F. Beugnet, C. Ollagnier, M.G. Pollmeier

**Affiliations:** 1 ClinVet, P.O. Box 11186, Universitas Bloemfontein 9321 Republic of South Africa; 2 Merial S.A.S. 29, avenue Tony Garnier 69007 Lyon France; 3 Merial S.A.S., PIPA, CRSV 1, allée des Cyprès 01150 Saint-Vulbas France

**Keywords:** *Dermacentor reticulates*, fipronil/amitraz/(S)-methoprene, imidacloprid/permethrin, ADVANTIX^®^, CERTIFECT^®^, *Dermacentor reticulatus*, fipronil/amitraz/(S)-méthoprène, imidaclopride/perméthrine, ADVANTIX^®^, CERTIFECT^®^

## Abstract

The sustained speed of kill against *Dermacentor reticulatus* of two topical combinations, one containing fipronil/amitraz/(S)-methoprene and the other, imidacloprid/permethrin, was evaluated in dogs. Two treated groups and one untreated control group of eight adult Beagle dogs each were randomly formed based on pre-infestation rates and bodyweight. Each treatment was administered topically once on Day 0, according to the recommended label dose and instructions for use. All dogs were infested with 50 adult unfed *D. reticulatus* starting on Day 1, then weekly, for a total of five weeks. While most studies determine tick efficacy at 48 hours (h), in this study, all remaining ticks were counted and categorized 24 h following each infestation. The numbers of ticks (living or dead) that remained attached on treated dogs were compared to those on the control animals. The percent reduction of attached ticks (disruption of attachment) at 24 h on dogs treated with fipronil/amitraz/(S)-methoprene remained above 92% for four weeks. The reduction of attached ticks at 24 h on dogs treated with imidacloprid/permethrin did not reach 80% during the entire study. The number of ticks attached at 24 h was significantly (p < 0.05) lower in the fipronil/ amitraz/(S)-methoprene group than in the imidacloprid/permethrin group in assessments on Days 2, 15, 22, 29 and 36. When assessing efficacy based upon live ticks on treated *versus* control dogs, fipronil/amitraz/(S)-methoprene 24 h efficacy was above 95% for four weeks, decreasing to 77.8% at Day 36. The 24 h efficacy of imidacloprid/permethrin ranged from 56.2% to 86.7% through Day 29, never achieving 90% throughout the study. The 24-hour efficacy of fipronil/amitraz/(S)-methoprene was significantly (p < 0.05) higher than imidacloprid/permethrin at all time points, including Day 36.

## Abstract (fr)

La rémanence et la rapidité d’action de deux antiparasitaires externes destinés au chien, l’un contenant fipronil/amitraz/(S)-méthoprène et l’autre imidaclopride/perméthrine, ont été évaluées. Deux groupes traités et un groupe témoin de chacun huit chiens de race Beagle ont été constitués après une infestation préalable par des tiques. Chaque traitement topique en pipette est appliqué au jour 0, selon les recommandations et instructions d’utilisation des laboratoires fabriquant. Tous les chiens sont infectés par 50 *D. reticulatus* adultes au jour 1, puis chaque semaine pendant cinq semaines. Alors que la plupart des études regardent l’efficacité anti-tiques à 48 heures (h), dans cette étude, les tiques restant sur les chiens ont été comptées et classées selon leur état (fixées ou non fixées, vivantes ou mortes, gorgées ou non gorgées) après 24 h de contact. Le nombre de tiques (vivantes ou mortes) qui restaient attachées sur les chiens traités a été comparé avec celui dénombré sur les chiens témoins non traités. Le pourcentage de réduction d’attachement (ou de perturbation d’attachement) à 24 h chez les chiens traités par la combinaison fipronil/amitraz/(S)-méthoprène est resté supérieur à 92 % pendant quatre semaines. La réduction d’attachement des tiques à 24 h chez les chiens traités par l’association imidaclopride/perméthrine n’a pas dépassé 80 % durant cet essai. Le nombre de tiques fixées sur les chiens à 24 h était significativement plus faible (p < 0.05) dans le groupe traité fipronil/amitraz/(S)-méthoprène que dans le groupe imidaclopride/ perméthrine aux jours 2, 15, 22, 29 et 36. Si l’on étudie l’efficacité acaricide par comparaison au nombre de tiques vivantes sur les chiens témoins, cette efficacité à 24 h est restée supérieure à 95 % pendant quatre semaines, puis a diminué à 77,8 % au jour 36 dans le groupe fipronil/amitraz/(S)-méthoprène. L’efficacité acaricide à 24 h dans le groupe traité imidaclopride/perméthrine a été comprise entre 56,2 et 86,7 % jusqu’au jour 29, ne dépassant jamais 90 %. L’efficacité acaricide à 24 h du groupe traité par l’association fipronil/amitraz/(S)-méthoprène a été significativement plus forte que dans le groupe imidaclopride/perméthrine à tous les points de comptage, jusqu’au jour 36.

Ticks and fleas are the primary ectoparasites infesting dogs (Pfister, 2011). *Dermacentor* tick species are present worldwide and can be vectors of disease for humans and animals (Berrada *et al*., 2009; Nicholson *et al*., 2010). In Western Europe, *Dermacentor reticulatus* is a common tick species affecting dogs and is the primary vector of *Babesia canis canis* (Beugnet & Marie, 2009). Effective control of tick infestations on dogs is based on several strategies, including avoidance of infested environments, environmental controls, and regular application of acaricides to the dog. Environmental control measures are often complex and have varying success rates. Tick infested environments are not easily identifiable; therefore avoidance is not always possible. Thus, regular topical applications of acaricides or combination products (*e.g.* insecticide/acaricide) are often used to control external parasite infestations in domestic animals (Brianti *et al*., 2010). Spot-on formulations of insecticides and/or acaricidal drugs provide a convenient method for external parasite control in dogs and cats.

In this study, two topical insecticide/acaricide combination products with labelled activity against ticks were chosen for comparison: the new topical combination fipronil/amitraz/(S)-methoprene in a dual chamber pipette (fipronil 10% w/v and (S)-methoprene 9% w/v in chamber one and amitraz 20% w/v in chamber two; CERTIFECT^®^ Spot-On Dog [registered trademark of Merial. All other marks are the property of their respective owners]) and imidacloprid/permethrin in a single chamber pipette (imidacloprid 10% w/v + permethrin 50% w/v; ADVANTIX^®^ [Europe; Bayer AG] or K9 ADVANTIX^®^ [United States; Bayer Animal Health]).

As a single entity, fipronil provides broad spectrum of activity against insects (including fleas and lice) and acari (including ticks and other mites). The new combination with amitraz was formulated and developed to significantly increase the speed of kill of fipronil on ticks, due to a potentiation effect provided by amitraz (Pfister, 2011; Prullage, Hair, et al., 2011). This new spot-on combination has been registered in 2011 in both the USA and Europe. Imidacloprid is an insecticide with no labelled activity against acari; thus, a combination product including permethrin was developed to create a product with a broadened range of efficacy. It is commercialized in the USA and in European countries since a few years.

Tick efficacy guidelines for approval in both EU and USA typically require efficacy ≥ 90% at 48-hour counts (Marchiondo *et al*., 2007). The purpose of the study was to assess the ability of each product to disrupt attachment of ticks and to assess the sustained speed of kill activity at 24-hour counts. Both rapid kill and disruption of attachment of newly acquired ticks would lower the numbers of engorged ticks seen, and potentially reduce the risk of transmission of many tick-borne pathogens. While rapid activity is desirable, it is equally important that rapid activity is sustained throughout the treatment interval. This study assessed the 24-hour activity against weekly infestations of *D. reticulatus* ticks, through Day 36.

## Materials and Methods

### Study Design

This study was a randomized, blinded, negative controlled comparative-efficacy study. All dogs were managed similarly and with due regard for their well-being. All were handled in compliance with local and Merial Institutional Animal Care and Use Committee approvals and in accordance with any applicable laws and regulations. Each dog was individually housed throughout the study.

From a pool of 28 healthy dogs, 24 dogs demonstrating the highest tick counts following a prequalification infestation were selected for use in this study. Dogs were allocated to one of eight replicates of three dogs each based on pre-treatment bodyweight, then the dogs within each replicate were randomly allocated to one of three groups of eight dogs each. Dogs used in this study hadn’t been treated with any ectoparasiticides in the preceding three months. Dogs were shampooed seven days prior to treatment with a non-insecticidal shampoo (Purl^®^ Shampoo, registered trademark of Kryon laboratories, South Africa).

Treatments were applied by weight, in accordance with approved-label directions on Day 0. The dogs were weighed to determine appropriate dosing. If the weight of any dog did not fall exactly on a whole pound, their weight was rounded up to the next whole pound. Dogs weighed between 27 and 53 lbs (12.2 to 24 kg).

Dogs in Treatment Group 1 remained untreated.

Dogs in Treatment Group 2 (Table I) were treated with the appropriate size of CERTIFECT^®^ Spot-On for dogs (in total volume applied on dogs: fipronil 6.26% w/v, amitraz 7.48% w/v and (S)-methoprene 5.63% w/v). The total volume was applied on the dog’s midline; half midway up the neck, half between the shoulder blades.

**Table I.** Dosage of dogs in the treated groups.

Treatment	Bodyweight range — lbs (kg)	Pipette size	Pipette volumes — ml
Certifect^®^ Spot-On for dogs	23–44 (10–20)	M	2.14
	45–88 (20–40)	L	4.28

Advantix^®^ spot-on for dogs	21–55 (10–25)	Red/55	2.5

Dogs in Treatment Group 3 (Table I) were treated with the appropriate size pipette of ADVANTIX^®^ spot-on for dogs (imidacloprid 10% w/v + permethrin 50% w/v). For treatment administration, the total volume was applied according to package instructions by parting the hair and applying directly on the skin in three spots along the midline from the top back of the shoulder blades to the base of the tail.

The *D. reticulatus* ticks used in this study were a laboratory strain, obtained from the ClinVet colony, free from known tick-borne pathogens and from a strain with no known resistance to any ectoparasiticide.

Dogs were infested with 50 *D. reticulatus* (50:50 sex ratio), for pre-treatment (Day -3) and for each subsequent infestation (Days 1, 7, 14, 21, 28 and 35). Each dog was sedated, and the ticks were gently deposited on the lateral aspect of the dog’s chest. Any remaining ticks were counted 24 hours after each infestation.

All personnel involved with evaluation of efficacy were unaware of treatment status of dogs in the study. Personnel with access to the treatment assignments were identified prior to the first treatment being administered, and blinding was maintained throughout the study.

### Specification of Study Variables

The ticks were categorized according to Table II. Categorization of the ticks allow calculation of the percent efficacy (killing effect), as well as the calculation of the attachment rate, in comparison with the untreated control group.

**Table II.** Categorization of ticks for counting and interpretation.

Category	General findings	Attachment status	Interpretation for prevention of attachment	Interpretation for killing effect (acaricidal)
1	Live	Free	Demonstrated	Not demonstrated
2	Live	Attached; unengorged	Not demonstrated	Not demonstrated
3	Live	Attached; engorged^1^	Not demonstrated	Not demonstrated
4	Dead	Free	Demonstrated	Demonstrated
5	Dead	Attached; unengorged	Not demonstrated	Demonstrated
6	Dead	Attached; engorged^1^	Not demonstrated	Not demonstrated

Adapted from Marchiondo *et al*., 2007.

1 Engorged tick: a tick with a conspicuous enlargement of the alloscutum that has blood in its digestive tract, as shown by squeezing/ crushing of the tick on white paper.

### Data Analysis

To measure disruption of attachment rates, total counts of ticks in categories 2, 3, 5, and 6 (Table II), *i.e.* all “attached” categories (live or dead), were transformed to the natural logarithm of (count + 1) for calculation of geometric means by treatment group at each time point.

To measure the killing effect (% acaricidal efficacy), the total counts of adult ticks in categories 1 through 3 and 6 were transformed to the natural logarithm of (count + 1) for calculation of geometric means by treatment group at each time point. The ticks in the three “Live” categories, as well as in the “Dead, attached, engorged” category, were interpreted as treatment failures. Their counts were combined and the total for each dog was used in the subsequent analysis.

For both analysis, percent reduction from the negative control group (Treatment Group 1) mean was calculated for Treatment Groups 2 and 3, if applicable, at every post-treatment time point using the formula [(C - T) / C] × 100, where C is the geometric mean for the negative control group and T is the geometric mean for Treatment Group 2 or 3. Treatment Group 2 was compared to each of the other treatment groups (Treatment Groups 1 and 3) using Analysis of Variance on log count. All testing was two-sided at the significance level α = 0.05.

## Results

### Disruption of Attachment

**Fig 1.**
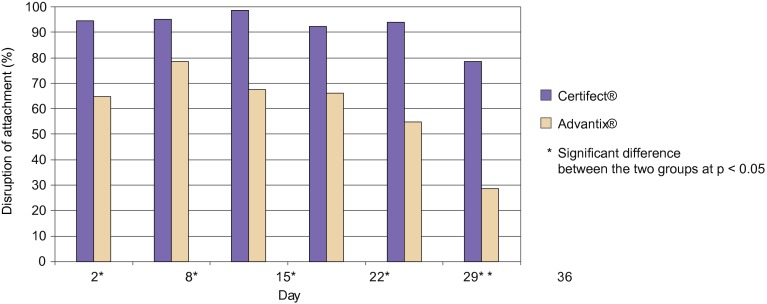
Disruption of attachment of *Dermacentor reticulatus* at 24-hour post-infestation.

The disruption of attachment is based on the counts of all attached ticks, live or dead, that are present at assessment, which in this study occurred at the 24-hour count (Table III, Fig. 1) (Prullage et al., 2011). The percentage of attachment at 24 hours was significantly higher (p < 0.05) in the control group than in the treated groups at all time points. The rate of attachment was significantly lower (p < 0.05) in the CERTIFECT^®^-treated group than the ADVANTIX^®^-treated group throughout the entire study. In the CERTIFECT^®^- treated group, 92.3% to 98.5% of ticks were prevented from attachment compared to the untreated group until Day 29. In the ADVANTIX^®^-treated group 54.7% to 78.4% of ticks were prevented from attachment compared to the untreated group until Day 29 (Table III).

**Table III.** Geometric means of *Dermacentor reticulatus* ticks counts by category at 24-hour post-infestation and percent of disruption of attachment and percent killing efficacy at 24-hour counts.

	Categories 2, 3, 5 and 6					Categories 1, 2, 3 and 6				
	Control	Certifect^®^		Advantix^®^		Control	Certifect^®^		Advantix^®^	
Day	Cnt.^1^	Cnt.	%^2^	Cnt.	%	Cnt.	Cnt.	%	Cnt.	%
2	23.46	1.28	94.6	8.26	64.8*	23.03	0.19	99.2	6.50	71.88*
8	24.75	1.21	95.1	5.34	78.4	23.42	0.00	100.0	3.11	86.78*
15	30.60	0.45	98.5	9.96	67.58*	30.49	0.22	99.3	8.93	70.78*
22	33.43	2.58	92.3	11.30	66.28*	33.18	1.59	95.2	8.90	73.28*
29	33.56	2.02	94.0	15.19	54.78*	33.43	1.65	95.1	14.63	56.28*
36	29.37	6.30	78.6	20.92	29.38*	29.46	6.55	77.8	21.56	27.38*

1 Cnt. = geometric mean tick count.

2 % = percent disruption of attachment at 24 hours for categories 2, 3, 5 and 6 or percent killing efficacy at 24-hour counts for categories 1, 2, 3 and 6.

* Significant difference between the two treatment groups at p < 0.05.

### Speed of Kill

**Fig 2.**
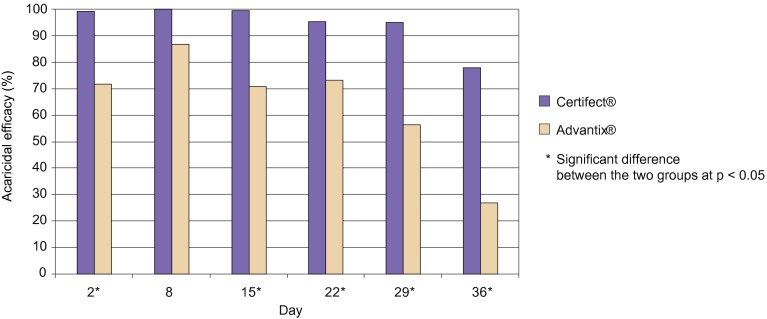
Comparative acaricidal efficacy at 24-hour tick counts.

The killing efficacy is shown on Table III and Fig. 2. The fipronil/amitraz/(S)-methoprene efficacy at 24- hour counts was above 95% through the four weeks following treatment, decreasing to 77.8% following the Day 35 challenge. The imidacloprid/permethrin 24 hours efficacy did not reach 90% efficacy at any assessment throughout the study, with killing efficacy through the four weeks ranging from 56.2% to 86.7% at 24-hour counts. The difference between the two treatment groups was significant at all time points including Day 36.

## Discussion

The tick counts in this study were conducted at 24 hours following each challenge, rather than 48 hours assessments. Previous studies have demonstrated that the efficacy of the imidacloprid/ permethrin combination at 48 hours is typically above 90% for three to four weeks or more, dependant upon the tick species studied (Doyle *et al*., 2005; Dryden *et al*., 2008; Tielemans *et al*., 2010). Another study performed against two tick species reported a rapid, but short-term activity of imidacloprid/permethrin causing a significant number of one of the tick species to remain unattached and repelled following infestation (Dryden *et al*., 2006). The quicker a product acts, the lower the probability that a tick will initiate its blood feeding and transmit pathogens. Short-term rapid activity is important, but pets can be exposed to ticks throughout the treatment interval, not just in the days immediately following treatment. Therefore, the duration of that rapid activity is key. By 24 hours after each infestation the fipronil/amitraz/(S)-methoprene combination showed disruption of attachment > 90% for a full month and efficacy > 95% against *D. reticulatus* for a full month, demonstrating both a rapid and most importantly a consistent performance throughout the treatment period. Such consistency would be important for pet owners, who do not want to see engorged ticks on their dogs. Moreover, it is expected that the disruption of attachment and efficacy provided by 24 hours would reduce the risk of transmission of many pathogens transmitted by ticks.
